# Dapagliflozin acutely improves endothelial dysfunction, reduces aortic stiffness and renal resistive index in type 2 diabetic patients: a pilot study

**DOI:** 10.1186/s12933-017-0621-8

**Published:** 2017-10-23

**Authors:** Anna Solini, Livia Giannini, Marta Seghieri, Edoardo Vitolo, Stefano Taddei, Lorenzo Ghiadoni, Rosa Maria Bruno

**Affiliations:** 1 Department of Surgical, Medical, Molecular and Critical Area Pathology, I-56126 Pisa, Italy; 2 Department of Clinical and Experimental Medicine, University of Pisa, Pisa, Italy

**Keywords:** Dapagliflozin, SGLT2-inhibitors, Thiazide diuretics, Endothelium, Flow-mediated dilation, Pulse wave velocity, Resistive index, Type 2 diabetes

## Abstract

**Background:**

Sodium-glucose cotransporter-2 inhibitors reduce blood pressure (BP) and renal and cardiovascular events in patients with type 2 diabetes through not fully elucidated mechanisms. Aim of this study was to investigate whether dapagliflozin is able to acutely modify systemic and renal vascular function, as well as putative mechanisms.

**Methods:**

Neuro-hormonal and vascular variables, together with 24 h diuresis, urinary sodium, glucose, isoprostanes and free-water clearance were assessed before and after a 2-day treatment with dapagliflozin 10 mg QD in sixteen type 2 diabetic patients; data were compared with those obtained in ten patients treated with hydrochlorothiazide 12.5 mg QD. Brachial artery endothelium-dependent and independent vasodilation (by flow-mediated dilation) and pulse wave velocity were assessed. Renal resistive index was obtained at rest and after glyceryl trinitrate administration. Differences were analysed by repeated measures ANOVA, considering treatment as between factor and time as within factor; Bonferroni post hoc comparison test was also used.

**Results:**

Dapagliflozin decreased systolic BP and induced an increase in 24 h diuresis to a similar extent of hydrochlorothiazide; 24 h urinary glucose and serum magnesium were also increased. 24 h urinary sodium and fasting blood glucose were unchanged. Oxidative stress was reduced, as by a decline in urinary isoprostanes. Flow-mediated dilation was significantly increased (2.8 ± 2.2 to 4.0 ± 2.1%, p < 0.05), and pulse-wave-velocity was reduced (10.1 ± 1.6 to 8.9 ± 1.6 m/s, p < 0.05), even after correction for mean BP. Renal resistive index was reduced (0.62 ± 0.04 to 0.59 ± 0.05, p < 0.05). These vascular modifications were not observed in hydrochlorothiazide-treated individuals.

**Conclusions:**

An acute treatment with dapagliflozin significantly improves systemic endothelial function, arterial stiffness and renal resistive index; this effect is independent of changes in BP and occurs in the presence of stable natriuresis, suggesting a fast, direct beneficial effect on the vasculature, possibly mediated by oxidative stress reduction.

## Introduction

The interaction between renal hemodynamics and BP is a complex issue, and this balance plays a key role in the sodium and water reabsorption by renal tubule. A raise in systemic BP influences intraglomerular pressure; an elevated renal interstitial hydrostatic pressure, direct consequence of systemic hypertension, rapidly inhibits sodium reabsorption by the proximal tubule, and the kidney responds by increasing sodium excretion; however, physiological mechanisms governing this adaptive response are only partially clarified [1]. Endothelium takes part in this complex mechanism: it has been suggested that pressure-mediated increases in vascular shear stress stimulate endothelial nitric oxide (NO) production, a signal able to reduce tubular sodium reabsorption [2, 3]; moreover, reactive oxygen and nitrogen species are key modulators of the pressure-natriuresis response [4].

Sodium-glucose co-transporter-2 (SGLT2) inhibitors are a novel class of glucose-lowering agents used in the treatment of type 2 diabetes (T2D). By inhibiting the transporter protein SGLT2 in the kidney, they reduce renal glucose reabsorption, promoting its urinary excretion. Unlike other oral antidiabetic drugs, the efficacy of these compounds is independent of insulin secretion and action [5], being exclusive function of plasma glucose levels and glomerular filtration rate.

Given their selective action on a dual transporter, SGLT2 inhibitors are supposed to increase natriuresis; however, several studies have denied an increased sodium (Na) excretion during SGLT2 inhibition [6–8]; a hypothetic up-regulation of other Na transporters has been suggested to explain this clinical finding. No doubt a precise quantitation of Na balance requires stabilization of its intake over several days; however, gross changes in serum Na concentration do not seem usual with chronic SGLT2 inhibition. At the nephron level, reduced Na reabsorption in the proximal segment increases Na delivery to the juxtaglomerular apparatus, thereby inhibiting the renin-angiotensin-aldosterone system (RAAS) [9]. In diabetic rats fed with a high-salt diet, SGLT2 inhibition prevents BP increase [10]; this effect may be counteracted by an activation of the RAAS, induced by excessive diuresis and volume depletion [11]. However, the mechanisms through SGLT2 inhibition reduce BP are not fully elucidated.

T2D patients show an impaired endothelial function, characterized by reduced NO availability, which can be demonstrated in peripheral arteries [12]; a further alteration in endothelial function has been shown after salt loading [13]. Endothelial dysfunction contributes to the development of hypertension, thereby promoting cardiovascular and renal damage [14]; therefore, improving endothelial function by restoring NO production with pharmacologic treatments may represent a useful tool and an important therapeutic goal in T2D [15]. The present study has been designed with the aim at evaluating whether, in T2D patients, an acute SGLT2 inhibition might modulate BP-related vascular parameters, like endothelium-dependent or independent vasodilation, arterial stiffness and renal vascular function by non invasive tests. As secondary endpoints, we explored RAAS and neuro-hormonal axis activation and oxidative stress as possible mechanism(s) concurring in explaining the vascular effects of dapagliflozin.

## Methods

### Subjects

Sixteen patients with T2D, consecutively recruited among those attending our diabetes outpatient’s clinic between January and September 2016 received dapagliflozin. Inclusion criteria were age between 40 and 70 years, BMI < 40 kg/m^2^, an adequate glucose control (HbA_1_c < 64 mmol/mol) reached with any oral anti-hyperglycemic drug, clinic BP values < 140/90 mmHg, treatment-naïve status for high BP. Exclusion criteria were an established hypertensive status, a moderate to severe renal function impairment (estimated glomerular filtration rate—eGFR < 60 ml/min/1.73 m^2^) and presence of clinically relevant cardiovascular/pulmonary/haematologic/hepatic disease, insulin treatment. Given the relatively unexpected significant BP reduction after only 2 days of dapagliflozin treatment, ten T2D patients were selected after the completion of the sixteen cases on the basis of the same inclusion criteria; they followed the same protocol but receiving hydrochlorothiazide (HCT), and served as control group, to test whether the results obtained in cases could be specifically related to the mode of action of dapagliflozin or, more extensively, to its diuretic and BP-lowering effect. This was conceived as a pilot study aimed at testing the aforementioned working hypothesis and planning larger studies in the future.

The nature and purpose of the study were carefully explained to all subjects before they provided their written consent to participate. The protocol was approved by the local Ethics Committee and registered in the Registry of the Italian Drug Agency (Agenzia Italiana del Farmaco, AIFA, #772/2015).

### Study design and protocol

When screened for eligibility, selected participants were advised to keep a daily Na intake of 92 mmol for the week preceding the study and until the end of the experimental protocol. Each subject was studied twice as outpatients: Visit 0, or baseline visit (day 0), and 2 days after a treatment with dapagliflozin 10 mg or HCT 12.5 mg QD (Visit 1, day 3); we chose 48 h on the basis of the pharmacokinetic properties of dapagliflozin [16]. In both visits, blood and urinary samples were collected for routine analyses, and determinations of plasma renin activity, aldosterone, norepinephrine, epinephrine and 24 h urinary electrolytes and 8-isoprostane; a complete non-invasive vascular evaluation, including flow-mediated dilation of the brachial artery (FMD), baseline and dynamic renal resistive index (RI), carotid-femoral pulse-wave velocity (PWV) and augmentation index (AIx) were also performed. All vascular tests were carried out by the same physician, with a long-lasting experience in this field (RMB).

### Endothelium-dependent and -independent vasodilation in the brachial artery

Endothelium-dependent response was obtained by FMD, as previously described [17]. A longitudinal ultrasound scan of brachial artery was performed by using a high-resolution ultrasound machine equipped with 10 MHz linear explorer (MyLab 25, ESAOTE Florence, Italy). After 1-min baseline recording, the cuff was inflated for 5 min at 300 ± 30 mmHg around the forearm and then deflated to induce reactive hyperaemia. Endothelium-independent vasodilation was obtained by the sublingual administration of 25 μg glyceryl trinitrate (GTN). Brachial artery diameter and flow velocity were continuously monitored by computerized edge detection system (Cardiovascular Suite; Quipu srl, Italy). FMD and response to GTN were calculated as the maximal percentage increase in diameter above baseline. Hyperemic stimulus was quantified by baseline and hyperemic shear rate (SR = 8 × mean flow velocity/brachial artery diameter) and hyperemic SR area under the curve (AUC). The intra-session (1 h apart) and inter-session (30 days apart) coefficients of variation were 7.6% (0.3–10.9) and 11.6% (2.1–13.2) in a group of 20 healthy volunteers [18].

### Renal resistive index

Three velocimetric measurements of the interlobar renal arteries (adjacent to the medullary pyramids) in the mesorenal area of each kidney were taken with a translumbar or anterior ultrasonographic approach. Renal RI was calculated in both kidneys according to the formula: (systolic peak velocity–end diastolic velocity)/systolic peak velocity and assessed both at baseline and 5 min after administration of GTN 25 μg. Dynamic Renal Resistive Index (DRIN) (%) was calculated as: (postGTN RI–baseline RI) × 100/baseline RI [19]. Intra-observer coefficient of variation was 2.1% for RI and 11.6% for DRIN measurements [19].

### Arterial tonometry

Arterial tonometry (SphygmoCor, AtCor Medical, Sidney, Australia) was performed according to the international recommendations [20]. Central BP was derived from radial pressure waveform by means of a validated transfer function and averaged on three measurements. Augmented pressure was calculated as the difference between the second and the first systolic peak, and AIx as the ratio between augmented pressure and pulse pressure (PP) and normalized at a heart rate of 75 bpm. Time to reflection was defined as the total travel time of the pulse-wave to the periphery and its return. Carotid-femoral PWV was calculated as the ratio of the surface distance between the two recording sites (direct distance * 0.8) and wave transit time. Three consecutive measurements were recorded and the median value was considered.

### Humoral parameters of renin-angiotensin and neurohormonal system

Plasma renin activity (PRA) and aldosterone were assayed by radioimmunoassay (DiaSorin, Saluggia, Italy). Plasma norepinephrine and epinephrine were assayed by high performance liquid chromatography.

### Urinary 8-isoprostanes

24-h urinary 8-isoprostane concentration was measured by a specific affinity sorbent. Urine samples were centrifuged to remove sediment (10 min at 800 g); aliquots of supernatants were incubated with 8-isoprostane affinity sorbent for 60 min according to the manufacturer’s instructions (Cayman Chemical, Ann Harbor, MI, USA).

### Statistical analysis

Statistical analysis was performed using NCSS 8 (NCSS, Kaysville, Utah, USA). Data were given as mean ± standard deviation when normally distributed, as median and interquartile range when non-normally distributed, and as count and percentages when discrete. Differences between groups at baseline were analysed by 2-sided paired Student’s t test for normally distributed continuous variables, Wilcoxon rank-sum test for not normally distributed continuous variables, or χ^2^ for categorical variables, as appropriate. Differences between Visit 0 and Visit 1 in the dapagliflozin and HCT treatment arms were analysed by repeated measures ANOVA, considering treatment as between factor and time as within factor. Bonferroni post hoc comparison test was also used. A post hoc sample size calculation was performed considering FMD as outcome variable and using the data collected in this study. A sample size of 24 patients was adequate to demonstrate the detected difference in FMD with a power of 0.82 and alpha 0.05.

A *p* value ≤ 0.05 was considered statistically significant.

## Results

The two groups were comparable for age (dapagliflozin 57 ± 9 vs HCT 60 ± 8 years, p = 0.29), sex (M/F 11/5 vs 7/3, p = 0.95) and BMI (30.5 ± 6.7 vs 28.5 ± 4.1 kg/m^2^, p = 0.26); HbA1c tended to be lower, even not significantly, in the HCT arm (56.0 ± 6.8 vs 49.2 ± 9.9 mmol/mol, 7.3 ± 2.8 vs 6.7 ± 3.1%). Table 1 shows the behaviour of serum variables in the study population. Acute treatment with dapagliflozin and HCT lowered clinic systolic BP values by a similar extent, whereas heart rate was significantly increased only in the HCT group (*p* = 0.04 for the interaction time*treatment).

**Table 1:** Behaviour of serum parameters before and after 2-day treatment with dapagliflozin or hydrochlorothiazide

	Dapagliflozin (n = 16)		Hydrochlorothiazide (n = 10)		p value (time × treatment interaction)
	Visit 0	Visit 1	Visit 0	Visit 1	
Plasma glucose (mmol/l)	7.96 ± 2.47	7.43 ± 2.1^b^	6.47 ± 1.16 (n = 6)	5.32 ± 0.88 (n = 6)	0.42
Haematocrit (%)	43.0 ± 2.7^b^	42.6 ± 3.1^b^	40.7 ± 4.1	40.4 ± 2.4	0.52
Total cholesterol (mmol/l)	4.62 ± 0.76	4.72 ± 1.09^b^	4.18 ± 0.59	3.87 ± 0.50	0.17
HDL cholesterol (mmol/l)	1.19 ± 0.23	1.24 ± 0.36	1.37 ± 0.24	1.29 ± 0.16	0.30
Triglycerides (mmol/l)	1.40 ± 0.55	1.50 ± 0.81	1.45 ± 0.31	1.16 ± 0.29	0.11
eGFR (ml/min/1.73 m^2^)	95.7 ± 12.8	93.7 ± 12.6	102.2 ± 6.1	97.8 ± 3.9	0.51
*s*-sodium (mEq/l)	141.0 ± 2.0	141.1 ± 1.2	139.3 ± 1.9	141.0 ± 2.6	0.13
*s*-potassium (mEq/l)	4.4 ± 0.3	4.3 ± 0.3	4.5 ± 2.1	4.2 ± 0.4	0.61
*s*-calcium (mg/dl)	9.6 ± 0.2	9.6 ± 0.3	9.5 ± 0.9	9.7 ± 0.9	0.03
*s*-chloride (mEq/l)	101.3 ± 2.9	100.3 ± 2.1	101.3 ± 2.9	102.0 ± 0.0	0.17
*s*-magnesium (mg/dl)	1.92 ± 0.19	2.04 ± 0.17^ab^	1.98 ± 0.33	1.90 ± 0.27	0.003
*s*-osmolarity (mOsm/l)	296 ± 5^b^	296 ± 2	290 ± 5	294 ± 7	0.04
Plasma renin activity (ng/ml/h)	0.50 ± 0.33	1.13 ± 0.97^ab^	1.09 ± 0.56	4.00 ± 0.06^a^	0.001
Aldosterone (pg/ml)	12.0 ± 4.7	14.3 ± 7.0	7.4 ± 4.6	3.9 ± 0.3	0.36
Norepinephrine (nmol/l)	414 [175]^b^	424 [73]	517 [419]	473 [413]^a^	0.18
Adrenaline (pmol/l)	25 [37]	25 [24]	41 [13]	40 [11]	0.86

^a^p < 0.05 vs V0

^b^p < 0.05 vs HCT (Bonferroni post hoc comparison)

At baseline, the two groups slightly differed by haematocrit and serum osmolarity (both higher in the dapagliflozin group). Humoral parameters did not vary in the two treatment arms, with the exception of serum magnesium concentration, which significantly rose (*p* = 0.003 for the interaction time*treatment) only in the dapagliflozin group. Both treatments induced a significant rise in plasma renin activity, greater for HCT group (*p* = 0.001 for the interaction time*treatment), but did not influence aldosterone levels, as well as the neuro-hormonal pattern, given that norepinephrine and epinephrine concentrations were unmodified.

Table 2 reports urinary parameters before and after treatment. As expected, 24 h diuresis significantly raised in both groups, whereas urinary glucose excretion increased and free water clearance declined only in dapagliflozin group. Noteworthy, free water clearance was strongly enhanced only in dapagliflozin treated patients, while Na fractional excretion was unmodified in both arms. 24 h urinary excretion of electrolytes, including magnesium, were unchanged by either treatment.

**Table 2:** Behaviour of urinary parameters before and after 2-day treatment with dapagliflozin or hydrochlorothiazide

	Dapagliflozin (n = 16)		Hydrochlorothiazide (n = 10)		p value (time × treatment interaction)
	Visit 0	Visit 1	Visit 0	Visit 1	
Diuresis (ml/24 h)	1400 [750]	2000 [750]^a^	1250 [150]	2100 [700]	0.47
*u*-sodium (mEq/24 h)	175 [107]	180 [57]	129 [96]	141 [173]	0.31
*u*-potassium (mEq/24 h)	60 [37]	64 [36]	56 [23]	59 [49]	0.10
*u*-calcium (mg/24 h)	220 [238]	158 [229]	250 [120]	93 [260]	0.47
*u*-chloride (mEq/24 h)	183 [113]	167 [79]	105 [121]	130 [163]	0.30
*u*-magnesium (mg/24 h)	70 [86]	72 [88]	100 [36]	78 [103]	0.34
*u*-osmolarity (mOsm/l)	687 ± 325	877 ± 230^b^	512 ± 76	377 ± 179	0.15
Glycosuria (mg/24 h)	406 [4224]	70,630 [40,110]^ab^	100 [84]	45 [135]	< 0.001
Osmolar clearance (ml/24 h)	2369 [3509]	5971 [3251]^a^	1982 [664]	2063 [2922]	0.28
Free water clearance (ml/24 h)	− 1119 [3441]	− 4221 [3549]^a^	− 757 [538]	36 [2222]	0.17
Na fractional excretion	0.89 ± 0.32	0.97 ± 0.34	0.62 ± 0.17	0.79 ± 0.07	0.72

Data are mean ± SD, or median [IQ]

*s* serum, *u* urinary

^a^p < 0.05 vs V0

^b^p < 0.05 vs HCT (Bonferroni post hoc comparison). The p value is for time × treatment interaction obtained by repeated measures ANOVA

**Fig. 1:**
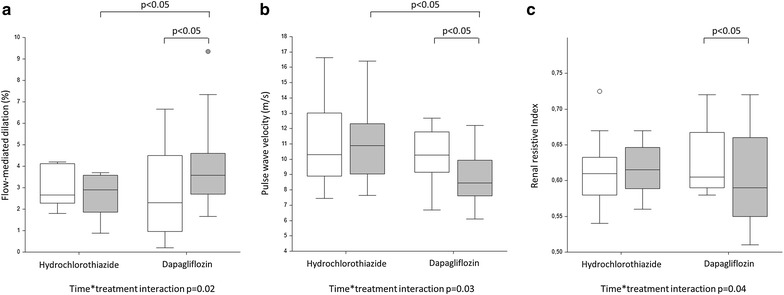
Box plots representing the behaviour of flow-mediated dilation (**a**), pulse wave velocity (**b**) and resting renal resistive index (**c**) in the study population before (in white) and after (in grey) 2-day treatment with dapagliflozin or hydrochlorothiazide

The behaviour of vascular variables is summarized in Table 3 and Fig. 1. Aortic PWV was significantly decreased by dapagliflozin but not by HCT (p = 0.03 for time*  treatment interaction), whereas central BP values and pressure augmentation variables were not significantly different. Treatment with dapagliflozin induced an increase in FMD (p = 0.02 for the time*treatment interaction), while the endothelium-independent brachial vasodilation did not vary. Furthermore, a significant increase in baseline brachial artery diameter was observed in the dapagliflozin arm (p = 0.03 for the time*treatment interaction), leading to a reduction in baseline and hyperemic shear rate. Finally, considering renal vascular variables, RI was significantly decreased after 2-day treatment with dapagliflozin (p = 0.04 for the time*treatment interaction). None of these parameters were influenced by HCT treatment.

**Table 3:** Behaviour of blood pressure and vascular parameters before and after 2-day treatment with dapagliflozin or hydrochlorothiazide

	Dapagliflozin (n = 16)		Hydrochlorothiazide (n = 10)		p value (time × treatment interaction)
	Visit 0	Visit 1	Visit 0	Visit 1	
Office systolic BP (mmHg)	130.6 ± 12.8	125.4 ± 11.2	137.2 ± 12.6	128.8 ± 11.2^a^	0.44
Office diastolic BP (mmHg)	75.3 ± 6.3	74.1 ± 6.9	76.1 ± 9.2	69.2 ± 7.3	0.06
Office pulse pressure (mmHg)	55.3 ± 11.2	51.3 ± 12.1	61.1 ± 10.4	59.6 ± 10.5	0.45
Pulse wave velocity (m/s) dir*0.8	10.1 ± 1.6	8.8 ± 1.6^ab^	11.0 ± 2.8	11.1 ± 2.6	0.03
Augmentation index (%)	30.2 ± 9.3	29.3 ± 11.2^b^	26.2 ± 5.3	22.1 ± 6.6	0.22
Augmentation index@75 (%)	26.3 ± 7.5	24.8 ± 10.1^b^	22.4 ± 5.8	19.6 ± 5.1	0.58
Central systolic BP (mmHg)	119.7 ± 10.6	116.0 ± 10.7	125.5 ± 11.1	115.2 ± 11.5	0.17
Central pulse pressure (mmHg)	44.2 ± 8.2	40.8 ± 11.5	48.4 ± 9.5	45.0 ± 10.3	0.99
Mean BP (mmHg)	94.4 ± 7.8	92.5 ± 6.6	97.5 ± 9.2	89.5 ± 7.5	0.11
Augmented pressure (mmHg)	13.6 ± 6.1	12.6 ± 7.5	12.8 ± 4.9	10.5 ± 5.1	0.44
Heart rate (bpm)	67.0 ± 12.0	65.5 ± 11.7^b^	67.1 ± 8.7	69.8 ± 9.3	0.04
Brachial artery diameter (mm)	4.29 ± 0.88^b^	4.46 ± 1.07^a^	4.52 ± 0.54	4.49 ± 0.51	0.03
Flow-mediated dilation (%)	2.81 ± 2.25	4.02 ± 2.09^ab^	2.99 ± 0.91	2.63 ± 1.01	0.02
Baseline shear rate (s^−1^)	199 ± 78^b^	140 ± 66^a^	279 ± 108	301 ± 118	< 0.001
Hyperemic shear rate (s^−1^)	787 ± 292	536 ± 337^ab^	927 ± 299	889 ± 399	0.05
Response to GTN (%)	6.36 ± 3.48	6.16 ± 2.87	4.11 ± 2.83	3.58 ± 2.89	0.85
Renal resistive index	0.62 ± 0.04	0.59 ± 0.05^a^	0.62 ± 0.05	0.62 ± 0.04	0.04
Dynamic renal resistive index (%)	− 6.10 ± 3.70	− 2.27 ± 4.39	− 2.96 ± 3.94	− 0.91 ± 3.8	0.43

^a^p < 0.05 vs V0

^b^p < 0.05 vs HCT (Bonferroni post hoc comparison). The p value is for time × treatment interaction obtained by repeated measures ANOVA

On univariate analysis, performed in the dapagliflozin group, absolute changes in PWV, FMD, and RI were not associated with changes in either brachial (r = 0.17, *p* = 0.74; r = 0.03, *p* = 0.90; r = − 0.42, *p* = 0.13 respectively) or aortic systolic BP (r = 0.08, *p* = 0.67; r = 0.01, *p* = 0.96; r = − 0.21, *p* = 0.47 respectively) or to changes in plasma and urinary variables. Systolic BP reduction (r = − 0.60, *p* = 0.02) and aldosterone increase (r = 0.70, *p* = 0.008) were significantly associated with increased osmole clearance.

Searching for a putative mechanism involved in the specific vascular effects of dapagliflozin, we found that 24 h urinary isoprostanes were significantly reduced by dapagliflozin (from 1659 ± 1029 to 1157 ± 663 pg/ml, *p* = 0.04) but not by HCT. Urinary 8-isoprostanes reduction was significantly associated with serum magnesium increase (r = − 0.81, *p* < 0.001) and urinary glucose excretion (r = − 0.57, *p* = 0.03).

## Discussion

The main finding of this pilot study, designed to explore the acute effects of dapagliflozin on systemic and renal vascular function, is a significant improvement in endothelial function, arterial stiffness and renal resistive index, occurring in the presence of stable blood glucose and natriuresis; a significant reduction in BP and oxidative stress was also found. A minor, albeit intriguing novel observation is a raise in serum magnesium concentrations, not coupled with a reduced urinary excretion. The study also confirms the lack of variation in the adrenergic tone; surprisingly, despite the claimed natriuretic effect of these compounds, natriuresis is unmodified too.

### Effect of dapagliflozin on systemic vascular function

We demonstrate here that dapagliflozin is able to induce a rapid improvement of conduit artery function, measured as FMD. These results are likely specific for SGLT2 inhibitors, since were not replicated in the HCT arm, as reported in previous studies [21]. Interestingly, this effect occurred in the presence of unchanged brachial artery diameter, hyperemic stimulus and response to GTN, indicating a selective positive effect of dapagliflozin on the endothelium, which was previously demonstrated only in animal models and after longer duration of treatments. Chronic therapy with SGLT2 inhibitors reverses vascular dysfunction by reducing oxidative stress in aortic rings from streptozotocin-treated rats [22] and from db/db rats [23], and chronically, but not acutely, enhances coronary vasodilation to NO donors in coronary arteries of diabetic mice [24]. Our study provides the first evidence in humans that dapagliflozin improves FMD and reduces oxidative stress; if confirmed in longer, placebo-controlled trials, the former effect might play a role in the reduction of cardiovascular risk induced by SGLT2 inhibitors [25, 26], while the latter could participate in the observed acute improvement in vascular function. Indeed, hyperglycemia-induced endothelial dysfunction is prevented by acute antioxidant administration [27]. Conversely, RAAS does not seem to mediate these acute vascular effects; the small increase in PRA we have observed with both treatments is conceivably a counter-regulatory response to the increased 24 h diuresis, as already reported [7]. Noteworthy, since both sympathetic activation [28] and hyperglycemia [29] may induce endothelial dysfunction, the presence of unchanged glucose and catecholamines levels after dapagliflozin treatment suggests a direct effect of dapagliflozin on the vascular endothelium. However, a sympatho-inhibitory effect of dapagliflozin cannot be definitely excluded since, after an acute reduction in BP, a baroreflex increase in catecholamines and heart rate might have been expected. The lack of HR increase in the dapagliflozin arm, at variance with HCT, reinforces the above-reported hypothesis. Dedicated studies, performed with more sophisticated and reproducible techniques [30], are needed to elucidate this crucial issue, though some neutral results were found in type 1 diabetic patients [31] and in animals [32].

In agreement with the reported restoration of endothelium-dependent vasodilation, the results regarding arterial stiffness are also of interest. The significant reduction in aortic PWV induced by dapagliflozin occurred after only 2 days of treatment and was independent of BP reduction, since was not observed in patients treated with HCT, in which a similar BP reduction was obtained. This finding suggests that dapagliflozin may act on the functional component of arterial stiffness, which is under the control of endothelium-mediated mechanisms especially in type 2 diabetes [12]. Cherney and coauthors demonstrated an effect of 8-week treatment with SGLT2 inhibitors on aortic stiffness in type 1 diabetic patients during hyperglicemic but not during euglycemic clamp [32]; however, these results are hardly comparable with those obtained in our study, for the different study population, treatment duration and experimental conditions.

In parallel with FMD findings, if a sustained reduction in PWV will be confirmed by longer trials, this may well explain a reduction in cardiovascular events [33]; in this view, studies with longer treatment duration are currently ongoing [34] to confirm whether a long-lasting effect on atherosclerosis and large artery stiffness does exist. The reduction in oxidative stress, that we explored as secondary outcome of our study, can be regarded as a unifying hypothesis of the positive effects on the vasculature.

### Effect of dapagliflozin on renal vasculature

Two-day dapagliflozin treatment, but not HCT, induced a significant decrease in renal RI. This index has been related to renal arteriolosclerosis and proposed as an integrated index of arterial compliance, pulsatility and downstream microvascular impedance [35]. However, there is an ongoing debate about the clinical significance and the physio-pathological meaning of this simple ultrasound parameter, since some evidence suggest that renal RI is influenced by systemic hemodynamics, rather than by renal vascular resistance [36]. Furthermore, recent data from our group suggest that in morbid obesity, a condition characterized by renal hyperfiltration and increased renal plasma flow, RI is directly correlated with renal plasma flow and inversely correlated with renal vascular resistance, and is reduced after bariatric surgery proportionally to renal plasma flow, suggesting that in these conditions RI is an index of hyperperfusion rather than of arteriolosclerosis [37, 38]. The effect of 8-week treatment with SGLT2 inhibitors on renal hemodynamics (measured by classical techniques, i.e. inulin and para-aminohippurate clearance) has been so far explored only in type 1 diabetic patients [6, 39], where empagliflozin was able to significantly reduce hyperfiltration and hyperperfusion, possibly restoring tubuloglomerular feedback and increasing afferent arteriolar resistance. Thus, it is conceivable that the significant, acute RI reduction induced by dapagliflozin is explained by the reduction in renal plasma flow exerted by SGLT2 inhibition, blocking proximal tubule glucose and sodium reabsorption, which leads to increased sodium delivery to the macula densa and restoration of tubuloglomerular feedback. The fast time course of the effect and the broadly unchanged central pulse pressure exclude an effect of renal arteriosclerosis, as well as reduced systemic pulsatility, as possible mechanisms of acute RI reduction.

The assessment of renal vascular function by static or dynamic ultrasound imaging has recently emerged as an appealing target for identifying subtle vascular alterations responsible for the development of diabetic nephropathy [40]. High RI has a negative prognostic value in diabetic patients in terms of progression of renal disease [41] and it is associated with higher all-cause mortality in patients with chronic kidney disease [42]; treatment with RAS blockers, drugs able to modify the clinical course of diabetic nephropathy, reduces RI by reducing renal plasma flow through vasodilation of the efferent arteriole [43]. The correction of renal hyperperfusion, which is adjunctive and complementary to RAAS-blockade, concurs to renal protection exerted by dapagliflozin [7, 44]. In the present study DRIN, calculated as the reduction in RI rapidly induced by sublingual administration of low-dose GTN, was increased, indicating a reduced vasodilation to nitrates. DRIN might represent a marker of renal vasodilating capacity and a predictor of microalbuminuria onset in type 2 diabetic individuals [19, 45]; in the present study, dapagliflozin effects on DRIN are probably masked by the above-described effects on renal plasma flow.

### Effect of dapagliflozin on electrolytes balance

A novel observation coming from our data is to characterize for the first time as dapagliflozin acutely acts as a purely “glucoretic” compound; in other words, beside glycosuria, it increases free water excretion without modifying the urinary electrolyte profile.

A common, and likely incorrect concept, is that SGLT2 inhibitors exert a beneficial natriuretic effect, even though few studies have so far measured natriuresis during SGLT2 inhibition in type 2 diabetes [7]. Recently, a transient increase in urinary sodium and chloride excretion has been reported with empagliflozin [7, 44]; we could not confirm this observation with dapagliflozin. The lack of variation in natriuresis, even after a 2-day treatment, supports the hypothesis of an increased expression and/or functional activity of other sodium transporters that might account for an immediately increased distal Na reabsorption. It is unlikely that the lack of change in natriuresis is attributable to the baseline small difference in serum osmolarity, more likely influenced by baseline difference in haematocrit.

Furthermore, acute variations in serum potassium levels were not observed in the present study. In a similar experimental setting, empagliflozin induces a modest, albeit significant increase in serum potassium levels [7, 44]. We cannot exclude that this different behaviour might be due to the different SGLT2 selectivity of the two drugs; however, this selective action on the free water pool can be regarded as a clinically advantageous effect respect to diuresis induced by classic thiazide compounds.

Another small mechanistic contribution of the present study is to show here that an increased serum magnesium level following SGLT2 inhibition, recently reported by an ample meta-analysis [45], is an immediate effect. Interestingly, magnesium in the high-normality range, is associated with a lower cardiovascular risk [46]. Furthermore, magnesium supplementation exerts favorable effects on endothelial function in individuals at risk for diabetes [47, 48], suggesting another possible mechanism through which SGLT2 inhibitors achieve vascular protection.

Some limitations of the present study should be acknowledged. First, this is a pilot study; though performed in a carefully selected population, the promising beneficial effects of dapagliflozin on endothelial function should be confirmed in larger cohorts and by randomized clinical trials. This is particularly relevant, since adjustment for known determinants of vascular function (i.e. brachial artery diameter for FMD), crucial for the interpretation of our findings [49], was not possible in the present study due to the small sample size. Second, before the described acute vascular effects can translate to an effective reduction of the cardiovascular risk, a demonstration of a long-lasting improvement in vascular function is necessary.

## Conclusions

In type 2 diabetic individuals, acute dapagliflozin administration has direct, beneficial effects on systemic and renal vasculature. These pleiotropic effects, which are conceivably BP- and glucose-independent, could represent a mechanism for cardiovascular protection for this drug class [50]. Furthermore, the rapid osmotic diuresis with neutral effects on electrolyte balance qualifies SGLT2 inhibitors as a valid, safe and intriguing alternative to thiazide diuretics in type 2 diabetic patients.

## Abbreviations

AUC: area under the curve
AIx: augmentation index
DRIN: dynamic renal resistive index
FMD: flow-mediated dilation
GTN: glyceryl trinitrate
HCT: hydrochlorothiazide
NO: nitric oxide
PP: pulse pressure
PRA: plasma renin activity
PWV: pulse-wave velocity
RAAS: renin–angiotensin–aldosterone system
RI: resistive index
SGLT2: sodium-glucose co-transporter-2
SR: shear rate
T2D: type 2 diabetes
